# Serological makers of rubella infection in Africa in the pre vaccination era: a systematic review

**DOI:** 10.1186/s13104-015-1711-x

**Published:** 2015-11-25

**Authors:** Mariam M. Mirambo, Mtebe Majigo, Said Aboud, Uwe Groß, Stephen E. Mshana

**Affiliations:** Department of Microbiology and Immunology, Weill Bugando School of Medicine, Catholic University of Health and Allied Sciences, P.O. Box 1464, Mwanza, Tanzania; Department of Microbiology and Immunology, Muhimbili University of Health and Allied Sciences, P.O. Box 65001, Dar es Salaam, Tanzania; Institute of Medical Microbiology, University Medical Centre Göettingen, Göttingen, Germany

**Keywords:** Rubella, Serological markers, Genotypes, Congenital rubella syndrome, Africa

## Abstract

**Background:**

Rubella infections in susceptible women during early pregnancy often results in congenital rubella syndrome (CRS). World Health Organisation (WHO) recommends that countries without vaccination programmes to assess the burden of rubella infection and CRS. However; in many African countries there is limited data on epidemiology of rubella infection and CRS. This review was undertaken to assess the serological markers and genotypes of rubella virus on the African continent in order to ascertain the gap for future research.

**Findings:**

A systematic search of original literatures from different electronic databases using search terms such as ‘rubella’ plus individual African countries such as ‘Tanzania’, ‘Kenya’, ‘Nigeria’ etc. and different populations such as ‘children’, ‘pregnant women’ etc. in different combinations was performed. Articles from countries with rubella vaccination programmes, outbreak data and case reports were excluded. Data were entered in a Microsoft Excel sheet and analyzed. A total of 44 articles from 17 African countries published between 2002 and 2014 were retrieved; of which 36 were eligible and included in this review. Of all population tested, the natural immunity of rubella was found to range from 52.9 to 97.9 %. In these countries, the prevalence of susceptible pregnant women ranged from 2.1 to 47.1 %. Rubella natural immunity was significantly higher among pregnant women than in general population (P < 0.001). Acute rubella infection was observed to be as low as 0.3 % among pregnant women to 45.1 % among children. All studies did not ascertain the age-specific prevalence, thus it was difficult to calculate the rate of infection with increase in age. Only two articles were found to report on rubella genotypes. Of 15 strains genotyped; three rubella virus genotypes were found to circulate in four African countries.

**Conclusion:**

Despite variations in serological assays, the seroprevalence of IgG rubella antibodies in Africa is high with a substantial number of women of childbearing age being susceptible to rubella infection. Standardized sero-epidemiological data in various age groups as well as CRS data are important to implement cost-effective vaccination campaigns and control strategies.

**Electronic supplementary material:**

The online version of this article (doi:10.1186/s13104-015-1711-x) contains supplementary material, which is available to authorized users.

## Findings

### Background

Rubella or ‘German measles’ is a mild viral disease caused by the rubella virus. Rubella is RNA virus in the family *Togaviridae* and is transmitted by droplets, direct contact or vertically from pregnant woman to the fetus [1]. The virus is worldwide distributed and is of public health concern due to its teratogenic effects. Infections in susceptible women during early pregnancy may results into multiple birth defects known as congenital rubella syndrome (CRS). Each year more than 100,000 children particularly in developing countries are born with CRS [2–4]. The CRS is mainly characterized by a triad of congenital heart diseases, congenital cataracts, and deafness; and many other defects [5].

Rubella is among many vaccine-preventable diseases; the main goal of vaccination is to reduce the incidence of rubella virus infection and CRS. In countries with vaccination programme especially in developed countries, the number of CRS cases have been markedly reduced [6, 7]. Despite the decrease in number of CRS cases worldwide, rubella remains a public health problem in Africa [3, 4]. Lack of vaccination programme in children contributes to increase in CRS cases because children usually harbour and spread the infection in community including susceptible pregnant women [8].

Despite high prevalence of congenital malformations in Africa [9, 10] few countries have introduced rubella vaccination in their national immunization programs to reduce incidence of acute rubella infections and CRS cases. World Health Organization (WHO) recommends that countries without national rubella vaccination programmes should assess the burden of rubella and CRS through sero-epidemiological surveys that may be implemented in parallel with measles surveillance [11]. However, there is limited data on epidemiology of rubella and CRS in Africa. The main objective of this review was to determine the gap of literatures based on WHO recommendations and accuracy of data to be used as baseline before rubella vaccination is introduced.

### Methods

Following PRISMA checklist (Additional file 1) systematic review was done. Systematic search for literature/original articles published in english focusing on rubella sero-epidemiology in Africa was performed using online database (PubMed/Medline, Embase, Popline, Global Health, Google Scholar and Web of Knowledge). The search was performed using terms; ‘rubella’ plus individual African countries like Tanzania, Kenya, Liberia, Nigeria etc., seroprevalence, pregnant women, adolescents, children in different combinations.

**Fig. 1:**
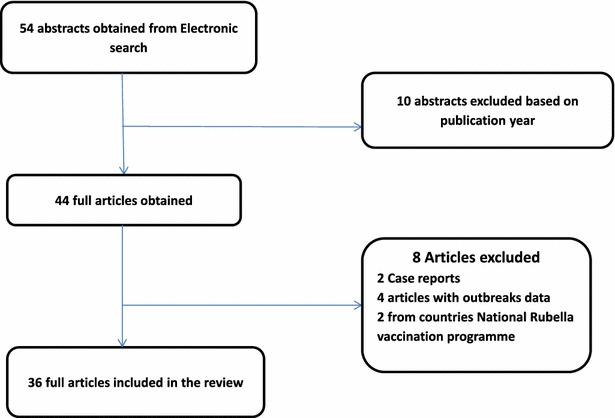
Flow chart showing articles and exclusion criteria

New links displayed in each abstract were followed and more abstracts were retrieved. Abstracts were carefully reviewed to exclude all articles published before 2002. Bibliographies of the retrieved articles were carefully reviewed and relevant articles published within the time frame were also retrieved. The search revealed 44 articles from 17 countries published between 2002 and 2014. Further analysis excluded; 2 case reports, 4 articles with outbreak data (WHO surveillance) and 2 articles from countries with national rubella vaccination programme as per WHO report (http://www.who.int/immunization/monitoring_surveillance/burden/vpd/surveillance_type/active/Rubella_map_schedule.jpg?ua=1) (Fig. 1).

### Data extraction and analysis

Eligible articles were reviewed independently by two authors. The data were recorded in excel sheet containing the subtitles such as region (country), study design, study population, age range, technique used, cut-off points, Immunoglobulin M (IgM) and Immunoglobulin G (IgG) seroprevalence, number of samples tested negative, whether the study was conducted in rural or urban settings, season, strain of circulating virus (if any), author and date of publication. Data were manually analysed to obtain proportions of natural immunity. Proportion test using STATA version 11 was done to establish statistical differences.

### Results

Of 36 articles reviewed, 20 (55.5 %) were conducted between 2007 and 2014. In these articles; population tested included pregnant women aged between 15 and 50 years, children aged from 0 to 18 years and general adult population aged between 19 and 62 years. The rubella natural immunity in these countries ranged from 52.9 % among pregnant women in Benin Nigeria to 99.3 % among adults in Uganda (Tables 1 and 2). High level of natural immunity was observed in population aged between 20 and 40 years.

**Table 1:** Summary of published articles regarding rubella in pregnant women in Africa between 2002 and 2014

Location/Country	Age	Sample size	IgG+	Cut off points	Trimester	Study period	References
Mwanza, Tanzania	15–44	Urban = 161 Rural = 181	Urban = 146 (90.6 %) Rural = 171 (94.5 %) Overall (92.7 %)	10 IU/ml	1 = 13 2 = 171 3 = 133	Nov 2012–May 2013	[28]
Ibadan south west (SW), Nigeria	15–42	273	244 (89.4 %)	10 IU/ml	NR	July 2010	[36]
Ibadan, Nigeria	15–39	159	109 (68.5 %)	20 IU/ml	NR	March–Oct 2002	[24]
Ilorin, Nigeria		92	78 (84.8 %)	0.55U/ml	1 = 23 2 = 68 1 = none	July–September 2009	[37]
SW Nigeria	19–44	90	86 (95.5 %)	>10 IU/ml	NR	Oct 2011–May 2012	[38]
Makurdi, Nigeria	18–36	534	NR	NR	1 = 534	Feb–July 2007	[26]
SE, Nigeria	19–30	345	329 (95.4 %)	NR	NR	June–Sept 2012	[39]
Benin, Nigeria	16–45	270	143 (52.9 %)	≥10 IU/ml	NR	NR	[27]
Zaria, Nigeria	19–45	Urban = 387 Rural = 43	Urban = 380 (98.2 %) Rural = 41 (95.3 %) Overall (97.9 %)	NR	NR	May 2007–Fe 2008	[40]
Borno, Nigeria	15–40	90	75 (83.3 %)	≥10 IU/ml	1 = 11 2 = 23 3 = 56	NR	[41]
Osogbo, Nigeria	15–40	200	175 (87.5 %)	>10 IU/ml	NR	March–June 2011	[42]
W. Sudan	25.7^a^	231	151 (65.4 %)	≥10 IU/ml	NR	Aug–Oct 2009	[43]
Khartoum state, Sudan	16–40	80	55 (68.7 %)	NR	NR	June 2012	[44]
Khartoum state, Sudan	16–47	500	465 (93 %)	15 IU/ml	NR	Nov 2008–March 2009	[45]
Maputo, Mozambique	15–30	Urban = 653 Rural = 321	Urban = 606 (92.8 %) Rural = 311 (96.8 %) Overall (94.1 %)	≥15 IU/ml	NR	Feb–April 2002	[46]
Ouagadougou, Burkina Faso	18–50	100	77 (77 %)	NR	NR	2006–2008	[47]
Burkina Faso	16–42	Urban = 132 Rural = 209	Urban = 125 (94.7 %) Rural = 193 (92.3 %) Overall (93.2 %)	≥10 IU/ml	NR	Dec 2007–March 2008	[48]
Kenya	15–46	Urban = 470	437 (92.8 %)	NR	NR	June–Dec 2005	[49]
Western cape, S. Africa	15–45	1200	1158 (96.5 %)	0.2 U/ml	NR	NR	[50]
Cameroon	15–40	211	186 (88.1 %)	>15 IU/ml	NR	April–July 2008	[51]
Benin	15–41	Rural = 283	266 (94 %)	NR	NR	July–September 2011	[52]
Total			90 % (52.9–97.9 %)				

*NR* not reported, *NA* not applicable

^a^Mean age

**Table 2:** Summary of published articles regarding rubella seroprevalence in general population in Africa between 2002 and 2014

Location/Country	Population	Age (years)	Sample size	IgG+	Cut off points	Study period	References
Oyo state, Nigeria	Reproductive age	15–45	230	215 (93.4 %)	NR	2002	[53]
Vom, Nigeria		20–65	96	87 (90.6 %)	NR	NR	[54]
Kenya	Children	4–20	498	398 (79.9 %)	NR	Feb–June 2005	[12]
Uganda	Adults	20–62	311	309 (99.3 %)	NR	NR	[55]
Bangui, Central Republic of Africa	Children	1 month–15	395	218 (55.1 %)	≥15 IU/ml	July–Dec2008	[13]
Algeria	Reproductive age	15–49	834	572 (68.6 %)	>10 IU/ml	March 2005–March 2007	[56]
Senegal	Reproductive age	15–45	3471	3127 (90 %)	>10 IU/ml	March–June 2002	[57]
Egypt	Women	6–45	Urban = 172 Rural = 426	Urban = 170 (98.8 %) Rural = 386 (90.6 %) Overall (96.3 %)	>15 IU/ml	NR	[58]
Lagos, Nigeria	HIV population	18–60	80	59 (73.8 %)	NR	April 2011–May 2012	[59]
Morocco	Reproductive age	15–39	Urban = 502 Rural = 465	Urban = 427 (85.1 %) Rural = 379 (81.5 %) Overall (83.3 %)	>8.6 IU/ml	2000	[60]
Total				84.8 % (55.1–99.3 %)			

*NR* not reported

**Fig. 2:**
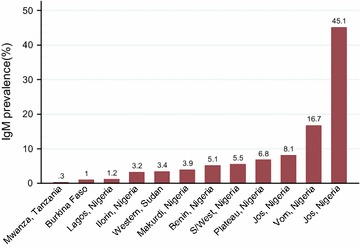
IgM seroprevalence in general population in different African countries. The highest prevalence is observed in Jos (45.1 %), Nigeria among children (1–10 years) and lowest is observed in Tanzania (0.3 %) among the pregnant women (15–44 years). Of 2039 tested for IgM, 143 (7 %) were positive

Of 36 articles reviewed, 22 (61.1 %) articles reported sero-prevalence of rubella-specific antibodies among pregnant women. The natural immunity of rubella among pregnant women was found to range from 52.9 % in Benin, Nigeria to 97.9 % in Zaria, Nigeria; implying that pregnant women susceptible to rubella infection in these countries range from 2.1 to 47.1 % based on different cut off points used. Overall, of 7215 pregnant women tested, 6494 (90 %, 95 % CI; 83.9–85.6) were found to have natural immunity compared to 6343 (84.8 %, 95 % CI; 89.2–90.7) of 7480 of general population tested (P < 0.001). Only 12 (33.3 %) articles reported IgM seroprevalence. In these articles, IgM seroprevalence was found to range from 0.3 % among pregnant women in Mwanza, Tanzania to 45.1 % among children in Jos, Nigeria (Fig. 2).

From few studies which determined seroprevalence in urban or in rural settings; the prevalence of rubella-specific IgG from urban settings ranged from 85.1 % in Morocco to 98.2 % in Zaria Nigeria (Tables 1 and 2) while in rural population it ranged from 81.5 % in Morocco to 96.8 % in Maputo, Mozambique. While, of the 22 articles which reported rubella-specific seroprevalence rates in pregnant women, only 6 articles categorized the study participants in relation to residence (rural or urban). No significant difference in natural immunity was observed between these two settings (Tables 1 and 2).

Regarding the techniques used in these studies all studies used Enzyme immunoassay (EIA)/enzyme linked immunosorbent assay (ELISA) techniques with significant variation of the cut off points of these assays. In addition, a total of 11 (30.5 %) studies did not specify the cut off points used.

Moreover, only two articles assessed rubella virus genotypes in Africa. In five countries where rubella virus were obtained and genotyped, it was revealed that at least three genotypes existed in Africa. Genotype 1E exists in Morocco and Sudan; 1G in Uganda, Sudan and Cote d’Ivore while genotype 2B exists in South Africa and Sudan. Of 15 strains genotyped between 2001 and 2010, 7 were typed as 1E, 5 as 2B and 3 as 1G genotypes. Results indicate that about 20 % of rubella virus strains circulating in Africa are non 1E and 2B.

### Discussion

Data from different African countries suggests that rubella virus is common. In addition these data indicate that there is significant number of susceptible women of childbearing age signifying the potential risk for giving birth to a child with CRS. High seroprevalence rates suggest that rubella virus is constantly circulating in Africa continent. Majority of population acquire infections in early childhood which accounts for high natural immunity in adolescence and among childbearing women [12–14].

The general rubella-specific IgG seroprevalence in pre-vaccination era in Africa is comparable with other regions in Southern America, India and Europe before vaccination [15–19]. However, the level of natural immunity in these studies is lower than the immunity currently reported in Europe; this might be due to on-going vaccination programmes in developed countries [20, 21].

Compared to the data from Europe there is a wide variation of rubella susceptibility in Africa indicating that transmission rates differ among regions. This could also be attributed to lack of standardized assays as confirmed in this review. Data from Europe are before 1970s while for Africa are of 2000s. This might possibly indicate higher population density variation worldwide.

Generally, no significant difference was observed for IgG seroprevalence between urban and rural settings in Africa. Similar findings have been observed in South America, India and Asia [18, 22].

As documented in other regions; higher IgM sero-prevalence rate was observed in children and adolescents than in adults, with the trend of IgM positivity decreasing with increase in age [14, 23–28]. This indicates high transmission rates during childhood emphasizing importance of vaccination in this age group [29].

There are little information available on genotypes of rubella viruses in Africa [30, 31]. Of ten genotypes known todate (1A, 1B, 1C, 1D, 1E, 1F, 1G, 2A, 2B and 2C) worldwide, 1E and 2B have been found to have a wide geographical distribution [29, 32, 33]. This might not represent a true picture in Africa because, of 15 strains genotyped between 2001 and 2010 in Africa; three (1E, 2B and 1G) genotypes were detected with 20 % of strains being non 1E and 2B [30, 31]. The rare genotype 1G which in Africa has been reported in Uganda has been previously observed in Israel, Europe and Brazil [34]. This necessitates the need for more phylogenetic studies in Africa which will be useful in monitoring genotypes changes in future.

Generally this review gives an overview of the current situation of rubella virus infection in Africa which may be useful for future control strategies. However, a number of challenges/drawbacks have been observed which accounts for limitations in some of the epidemiological information. These challenges include: different serological assays utilizing different reagents and cut off points have been used, making a comparison between the studies difficult. In addition, some of the articles did not document cut-off points for IgG seroprevalence. Most of the articles did not assess both IgM and IgG seroprevalence at one point in time. This might cause inaccurate information about susceptible individuals in some studies because some of the IgG-negative individuals were probably IgM positive. The magnitude of IgM seropositivity might have been overestimated due to high false positive rate of IgM assays. Therefore the data provided in this review underscore the need for standardizing surveillance in Africa for rubella virus infection. Majority of the articles did not indicate whether the participants were from rural or urban settings. Therefore it was difficult to assess the level of immunity on these two populations in the continent. Of more important, age specific seroprevalence and incidence rates were not reported in majority of the articles. Therefore, this information is not very clear in Africa making the basis of age limit for vaccination questionable. Another, information which is not clear regarding rubella infection in Africa is seasonality as none of the articles reviewed investigated on this aspect. Lastly, none of the studies assessed the sero-conversion rate during the course of the pregnancy hence it is difficult to estimate the potential risk for CRS in Africa. In addition, the data of the magnitude of CRS was not documented emphasizing the need for surveillance of CRS in Africa [35].

### Conclusion

There are few studies in Africa which have investigated on molecular epidemiology of rubella virus, therefore little is known regarding genotypes of rubella virus strains circulating in Africa. In addition very few studies have compared epidemiology of rubella virus between urban and rural settings. Finally, descriptions of assay/techniques used were very poor. There is a need to follow WHO guidelines when conducting epidemiological research so that data can easily be pooled and help in policy formulation.

## Additional file

10.1186/s13104-015-1711-x PRISMA checklist.

## Abbreviations

CRS: congenital rubella syndrome
ELISA: enzyme linked immunosorbent assay
EIA: enzyme immunoassay
IgG: immunoglobulin G
IgM: immunoglobulin M
WHO: World health organisation
