# Erratum to: Serological makers of rubella infection in Africa in the pre vaccination era: a systematic review

**DOI:** 10.1186/s13104-016-2129-9

**Published:** 2016-07-05

**Authors:** Mariam M. Mirambo, Mtebe Majigo, Said Aboud, Uwe Groß, Stephen E. Mshana

**Affiliations:** Department of Microbiology and Immunology, Weill Bugando School of Medicine, Catholic University of Health and Allied Sciences, P.O. Box 1464, Mwanza, Tanzania; Department of Microbiology and Immunology, Muhimbili University of Health and Allied Sciences, P.O. Box 65001, Dar es Salaam, Tanzania; Institute of Medical Microbiology, University Medical Centre Göettingen, Göttingen, Germany

## Erratum to: BMC Res Notes (2015) 8:716 DOI 10.1186/s13104-015-1711-x

Unfortunately the original version of this article [1] contained an incorrect reference.

Reference number 45 was a duplicate of reference number 43. This should have read:

Adam O, Makkawi T, Kannan A, Osman ME. Seroprevalence of rubella among pregnant women in Khartoum state, Sudan. East Mediterr Health J 2013;19:812–15.
